# Real-Time Emission and Exposure Measurements of Multi-walled Carbon Nanotubes during Production, Power Sawing, and Testing of Epoxy-Based Nanocomposites

**DOI:** 10.1093/annweh/wxac015

**Published:** 2022-03-17

**Authors:** Maria Hedmer, Karin Lovén, Johan Martinsson, Maria E Messing, Anders Gudmundsson, Joakim Pagels

**Affiliations:** NanoLund, Center for Nanoscience, Lund University, 22100 Lund, Sweden; Occupational and Environmental Medicine, Department of Laboratory Medicine, Lund University, SE-22100 Lund, Sweden; Department of Occupational and Environmental Medicine, Region Skåne, SE-22381 Lund, Sweden; NanoLund, Center for Nanoscience, Lund University, 22100 Lund, Sweden; Ergonomics and Aerosol Technology, Department of Design Sciences, Lund University, SE-22100 Lund, Sweden; Medical Radiation Physics, Department of Translational Medicine, Lund University, SE-22100 Lund, Sweden; NanoLund, Center for Nanoscience, Lund University, 22100 Lund, Sweden; Solid State Physics, Department of Physics, Lund University, SE-22100 Lund, Sweden; NanoLund, Center for Nanoscience, Lund University, 22100 Lund, Sweden; Ergonomics and Aerosol Technology, Department of Design Sciences, Lund University, SE-22100 Lund, Sweden; NanoLund, Center for Nanoscience, Lund University, 22100 Lund, Sweden; Ergonomics and Aerosol Technology, Department of Design Sciences, Lund University, SE-22100 Lund, Sweden

**Keywords:** aethalometer, black carbon, elemental carbon, manufactured nano-object, monitoring, surface contamination

## Abstract

**Objectives:**

The use of manufactured nanomaterials is increasing globally. Although multi-walled carbon nanotubes (CNTs) are used in a wide range of applications, only limited data are available on emissions and exposures during CNT composite production. No exposure data using portable aethalometers in the personal breathing zone (PBZ) to monitor occupational exposure to CNTs have yet been published. The aim of this study was to characterize emissions of and exposures to CNTs during CNT composite production, sawing, and shear testing. We also investigated whether real-time aethalometer measurements of equivalent black carbon (eBC) could be used as a proxy filter sampling of elemental carbon (EC). The presence of CNTs as surface contamination in the production facility was monitored since this could contribute to airborne exposure.

**Methods:**

During CNT composite production in an industrial setting including both chemical and manufacturing laboratories, different work tasks (WTs) were studied with a combination of direct-reading instruments (aethalometer, aerodynamic particle sizer, condensation particle counter) and filter-based methods. Measurements were performed to monitor concentrations in the emission zone (EZ), PBZ, and background zone. The filter samples were analysed for EC and fibre concentration of CNTs using scanning electron microscopy (SEM). Additionally, surfaces in the facility were tape sampled for monitoring of CNT contamination, and analysed with SEM.

**Results:**

Clear eBC peaks were observed in the PBZ during several WTs, most clearly during open handling of CNT powder. Power sawing emitted the highest particle number concentration in the EZ of both nanoparticles and coarse particles, but no individual airborne CNTs, agglomerates, or aggregates were detected. Airborne CNTs were identified, for example, in a filter sample collected in the PBZ of a worker during mixing of CNT epoxy. The airborne CNT particles were large agglomerates which looked like porous balls in the SEM images. Significant EC exposures were found in the inhalable fraction while all respirable fractions of EC were below detection. The highest inhalable EC concentrations were detected during the composite production. No significant correlation was found between inhalable EC and eBC, most likely due to losses of large EC containing particles in the sampling lines and inside the eBC monitor. In total, 39 tape samples were collected. Surface contamination of CNTs was detected on eight surfaces in the chemical and manufacturing laboratories, mainly in the near-field zone. Elongated CNT-like features were detected in the sawdust after sawing of CNT composite.

**Conclusions:**

Characterization of a workplace producing CNT composite showed that open handling of the CNT powder during weighing and mixing of CNT powder material generated the highest particle emissions and exposures. The portable direct-reading aethalometer provided time-resolved eBC exposure data with complementary information to time-integrated EC filter samples by linking peak exposures to specific WTs. Based on the results it was not possible to conclude that eBC is a good proxy of EC. Surface contamination of CNTs was detected on several surfaces in the near-field zone in the facility. This contamination could potentially be resuspended into the workplace air, and may cause secondary inhalation exposure.

**What’s Important About This Paper?**

This study demonstrates that a portable direct-reading aethalometer can be used to measure carbon nanotubes (CNTs) in workers’ breathing zones, providing time-resolved equivalent black carbon exposure data as complementary information to the time-integrated filter sample. This method addresses the issue that aethalometer results underestimate the mass concentration of CNTs arising from the different optical properties of CNTs and other elemental carbon materials. Use of an aethalometer with time-integrated filter sample can enhance exposure assessment for CNTs.

## Introduction

The industrial use of novel manufactured nanomaterials with enhanced or completely new and unique properties is increasing globally. In recent decades, multi-walled carbon nanotubes (CNTs) have been used in a wide range of applications due to their superior mechanical strength and flexibility. CNTs are fibre shaped with a high aspect ratio, and show a high diversity due to differences in number of walls, diameter, length, chiral angles, chemical functionalization, purity, stiffness, and bulk density (Hedmer *et al.*, 2013). CNTs are added as fillers for reinforcement of composites (Jin *et al.*, 2015; Ding *et al.*, 2017; Ogura *et al.*, 2019). One type of composite, epoxy composite, is widely used due to its extraordinary strength, stiffness, and chemical resistance (Ging *et al.*, 2014).

The existing literature includes a few earlier studies of emission and exposure in connection to CNT composite production (Cena and Peters, 2011; Fleury *et al.*, 2013; Thompson *et al.*, 2015; Kato *et al.*, 2017), as well as studies of machining processes such as sanding and sawing of CNT composites (Bello et al., 2009, 2010; Cena and Peters, 2011; Wohlleben *et al.*, 2013; Ding *et al.*, 2017; Kuijpers *et al.*, 2019; Ogura *et al.*, 2019). There is high-quality evidence that workers are occupationally exposed to CNTs during production of CNTs, mainly in handling tasks such as pouring, weighing, mixing, harvesting, extruding, sonication, and packaging of CNT powder or liquid suspensions (Debia *et al.*, 2016). More data regarding occupational exposure in the later stages of the life cycle, for example during CNT composite production, are needed for realistic risk assessments of CNTs performed in full-scale industrial settings instead of laboratory experiments (Thompson *et al.*, 2015; Kuijpers *et al.*, 2019).

CNT emissions and exposures are typically collected with filter-based methods for subsequent analysis of elemental carbon (EC, as an indirect determination of CNTs), as well as identification or counting with electron microscopy. Occupational exposure limits have been proposed for these methods: 1 µg m^−3^ based on the respirable fraction of EC (NIOSH, 2013), and 0.01 fibre cm^−3^ for fibrous nanomaterials with high aspect ratios (>3:1 and length >5 µm; BSI, 2007). Filter-based methods result in time-integrated concentrations without information about peak exposures.

Reports in the literature state that equivalent black carbon (eBC) is a good proxy for EC since there is a strong correlation between the two (Allen *et al.*, 1999; Hashimoto *et al.*, 2013; Kim *et al.*, 2016). Mass concentrations of eBC can be derived from attenuation measurements at a wavelength of 880 nm using an aethalometer. This method has a high selectivity for carbonaceous aerosols such as EC and CNTs, as most other materials absorb much less at this wavelength. With aethalometers, it is possible to get time-resolved data (approximately in the range of minutes, depending on concentration) showing, for example peak exposures. Previously, eBC measurements have been used in workplaces to detect CNTs in the emission zone (EZ) or background zone (BGZ) (Han *et al.*, 2008; Lee *et al.*, 2010; Hashimoto *et al.*, 2013; Ji et al., 2013, 2015; Kim *et al.*, 2016). Thus, eBC may be used as a proxy for EC; however, the mass concentration of CNTs may be underestimated, as CNT materials may have different optical properties compared with other common EC materials such as diesel soot (Hashimoto *et al.*, 2013).

The objective of this study was to characterize CNT emission and exposure during downstream use such as nanocomposite production, machining by power sawing, and short-beam shear testing. Airborne measurements in the personal breathing zone (PBZ) as well as in the EZ near the expected emission source of CNTs were performed in an industrial setting. The parallel measurements in the PBZ were evaluated to study if eBC (personal real-time aethalometer measurements) could be a proxy for EC (time-integrated filter sampling) giving detailed exposure information about specific work activities, such as peak exposures. We also wanted to monitor if the exposure markers [eBC, EC, and scanning electron microscopy (SEM) samples] could be used to estimate occupational exposure to CNTs. An additional aim was examine the presence of CNTs deposited on surfaces and to monitor potential for secondary inhalation exposure via resuspension.

## Methods

### Industrial setting

In this study, we monitored emissions and exposures of CNTs at a downstream user where CNT materials were added during production of nanocomposites to increase the strength of the material, and where machining and testing of the CNT composites were performed. The nanocomposites produced could potentially be used in aircrafts or in joints of composites to metal in aircraft and ships.

The chosen company used different types of engineering control, such as fume hoods and local exhaust ventilation during production and testing of CNT composites (Table 1). Three workers, A, B, and C, performed the CNT work, and used negative-pressure half-face respirators with a particulate filter (P3) and a vapour cartridge (ABEK1) together with latex gloves and cotton lab coats. A fourth worker, D, worked with cleaning inside the chemical and manufacturing laboratories without respiratory protection.

**Table 1.** WTs performed during the sampling campaign, engineering controls used at the company, and condition of the CNTs. The numbers in the first column correspond to the numbers in Fig. 1.

No.	WT	Location	Worker	Engineering controls	Use of CNTs
1	Electrophoretic deposition (WT 1)	Chemical laboratory	A	Fume hood	Dispersed in liquid
2	Direct mixing of CNT with epoxy (WT 2)	Chemical laboratory	B	Fume hood	As powder
3	Vacuum infusion of CNT epoxy (WT 3)	Manufacturing laboratory	A, B	Ventilated oven	Dispersed in liquid
4	Preparation and vacuum infusion of epoxy (WT 4)	Chemical and manufacturing laboratories	A	Fume hood, ventilated oven	Coated on carbon fibre fabrics
5	Power sawing (WT 5)	Manufacturing laboratory	B	Local exhaust ventilation	Solid epoxy composite
6	Short-beam testing (WT 6)	Manufacturing laboratory	C	Local exhaust ventilation, curtain	Solid epoxy composite
7	Cleaning (WT 7)	Chemical and manufacturing laboratories	D	N/A^*a*^	N/A

^*a*^Not applicable.

Below follows a description of the CNT raw materials used, the laboratories and the work tasks (WTs) performed at the company during the sampling campaign (Table 1).

#### Multi-walled CNT raw materials

The company used the following commercially available CNTs in the production of nanocomposites: plain multi-walled CNT powder (Nanocyl, Belgium), and COOH functionalized multi-walled CNT as powder and as aqueous suspension (Nanolab, USA). CNTs were handled in pure powder form, dispersed in liquid, coated on carbon fibre fabrics, or embedded in epoxy composite.

#### Chemical and manufacturing laboratories

**Figure 1.**
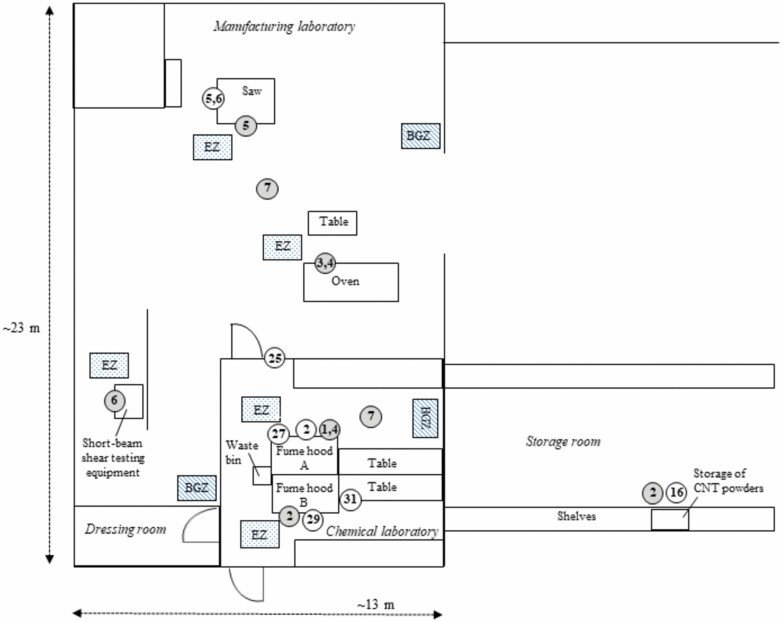
Schematic drawing of the facility at the second manufacturer (downstream user) of CNTs. The numbers in grey circles correspond to the numbers in Table 1 and show where the WTs were performed in the company. The numbers in white circles correspond to tape samples where surface contamination of CNTs were detected.

The layout of the facility is given in Fig. 1. The chemical laboratory had an area of 68 m^2^ and an air volume of 236 m^3^, and the manufacturing laboratory had an area of 260 m^2^ and an air volume of 950 m^3^. The laboratories had general and process ventilation systems such as fume hood and local exhaust ventilation. The chemical laboratory had two fume hoods where different WTs were carried out. The measurements in the EZ were performed inside these fume hoods. The manufacturing laboratory was equipped with an oven, a worktable, a sawing stand, and equipment for short-beam shear testing. The general housekeeping of the laboratories was considered to be on a basic level.

#### Work tasks

A description of the different WTs is presented below (see Table 1 and Fig. 1 for the positions where these WTs were performed in the facility). During the sampling campaign two different types of CNT composites were produced. A schematic overview of the production and testing steps at the manufacturer is given in Supplementary Fig. S1 (available at *Annals of Work Exposures and Health* online). Measurements were performed during production of CNT composite consisting of (i) CNT-coated carbon fibre fabrics and (ii) CNT mixed with epoxy. Parts of the different production steps were included in the production process for each specific CNT composite. For type (i), the CNT-coated carbon fibre fabrics were produced and then embedded in epoxy (WTs 1 and 4). For type (ii), CNTs were instead mixed with epoxy (WTs 2 and 3). For each WT, a detailed logbook was written by one of the researchers carrying out the study. The logbook had a time-resolution of minutes, and was used extensively in the data evaluation of this study.

##### Electrophoretic deposition (WT 1)

A suspension containing CNTs (NanoLab) was diluted with water in a fume hood in the chemical laboratory. The resulting suspension was moved to a second fume hood and stirred for 10 min. The suspension was then moved back to the first fume hood where the equipment used for the electrophoretic deposition (EPD) process was located. The CNT solution was poured into the EPD container. A sheet of carbon fibre fabric was placed in the EPD container and an electric field was applied for 10 min to coat the fabric with CNTs. The sheet was then placed in the fume hood to dry, and the equipment was cleaned and wiped dry. Two cycles of EPD were included in the measurements. The CNT-coated sheets were then used in WT 4.

##### Mixing of CNT epoxy resin (WT 2)

Plain CNT powder (Nanocyl) was handled openly in the manufacturing laboratory by scooping the powder from a bag into a paper cup. The powder was weighed in one of the fume hoods in the chemical laboratory and then poured into a metal can containing an epoxy hardener. The can was placed in an ice bath and sonicated for 30 min with 10 s pulses. After the sonication, epoxy base was added to the mixture and the can was placed in the ice bath again. The epoxy mixture was stirred for another 20 min, after which the stirrer was wiped off. The CNT epoxy resin was then used in WT 3.

##### Vacuum infusion process of CNT epoxy (WT 3)

The can with CNT epoxy resin was moved from the chemical laboratory and placed in the heated, ventilated oven (80°C) in the manufacturing laboratory. A prepared mould containing several layers of carbon fibre fabric was placed in the oven. CNT epoxy mixture was then driven into the reinforcement material in the mould at a low vacuum pressure.

##### Preparation and vacuum infusion of epoxy (WT 4)

Sheets of dried CNT-coated carbon fibre fabrics from the EPD (WT 1) were cut and placed in a mould together with untreated carbon fibre fabrics in one of the fume hoods in the chemical laboratory. A bagging film was placed at the top of the mould with sealant tape. The mould was then moved to the manufacturing laboratory, connected to vacuum and placed in a heated, ventilated oven (80°C) in the manufacturing laboratory. The vacuum system was used to drive an epoxy mixture was driven into the CNT reinforcement material in the mould.

##### Power sawing of CNT composite (WT 5)

A sheet of CNT composite material (size ~25 × 25 cm) was sawn into smaller pieces (*N* = 35) with a circular power saw in the manufacturing laboratory. A local exhaust ventilation was placed close to the saw blade during the sawing.

##### Short-beam shear testing of CNT composite (WT 6)

Small pieces (size ~3 × 10 cm) of CNT composite material (*N* = 15) were short-beam shear tested with specific testing equipment to study the performance of the composite materials. In the test, a piece of the composite material was bent to find the load at which the material failed. A plastic curtain shielded off the testing equipment from the manufacturing laboratory, and local exhaust ventilation was placed inside the shielding.

##### Cleaning of the chemical and manufacturing laboratories (WT 7)

Routine cleaning of the work areas, equipment, and floors in the chemical and manufacturing laboratories was performed with a vacuum cleaner.

### Workplace monitoring

#### Monitoring strategy

Air sampling was carried out in the PBZ, EZ (near-field), and BGZ (far-field). Both filter-based methods and direct-reading aerosol instruments were used to measure emissions and exposures during the CNT work (WTs 1–7). The measurements were carried out during four consecutive workdays. Workers wore the personal samplers at the lapels of their lab coats. The EZ samples were co-located with instrument inlets positioned together, within approximately 0.1–0.2 m from the potential source of a specific WT. The BGZ station with samplers and direct-reading aerosol instruments was at least 3 m away from the potential particle source. EC mass concentration, eBC, and number concentration of CNT-containing particles were sampled in parallel and used as exposure metrics. Both respirable and inhalable fractions of EC and CNT-containing particles were collected in parallel for comparison. An overview of the monitoring strategy is presented in Table 2.

**Table 2.** Monitoring strategy for each WT at the company.

Measuring location	Filter-based methods	Direct-reading instruments
PBZ	EC_INH_, SEM_INH_	eBC_DRI_
EZ	EC_INH_, EC_RESP_, SEM_INH_, SEM_RESP_	APS, CPC
BGZ	EC_INH_	APS, BC_DRI_, SMPS

eBC_DRI_, equivalent mass concentration of black carbon measured by aethalometer; EC_INH_, inhalable elemental carbon mass concentration; EC_RESP_, respirable elemental carbon mass concentration; SEM_INH_, inhalable number concentration of CNT-containing particles; SEM_RESP_, respirable number concentration of CNT-containing particles.

Deposition of airborne CNT-containing particles can result in both near-field and far-field surface contamination (Schneider *et al.*, 2011). Tape samples were therefore collected from surfaces related to the production and testing of CNT composites in the near-field zone of the exposure source (<1 m). To survey the scope of the surface contamination of CNTs in the facility, we also sampled surfaces in the far-field zone (>1 m), specifically the dressing room, office, and corridor outside the chemical and manufacturing laboratories.

#### Direct-reading instruments

##### Monitoring of eBC

Two portable aethalometers (microAeth^®^ model AE51 AethLabs, USA) were used in the study. An aethalometer is a real-time filter-based technique which measures the rate of change in attenuation of transmitted light due to continuous collection of aerosol particles on a filter (AethLabs, 2016). A wavelength of 880 nm was used, and the light attenuation was transformed to an eBC mass concentration using the standard settings of the instrument. The instrument is sensitive to other carbonaceous materials such as carbon blacks, soot, and EC in addition to CNTs (Han *et al.*, 2008; Lee *et al.*, 2010; Hashimoto *et al.*, 2013). Based on our observations onsite we assumed that no other EC sources were present at the workplace, and hence that the measured mass concentration of eBC originated from CNTs. The time-resolution was 1 min, the flow rate was 150 ml min^−1^ and the total suspended particulate matter fraction (no pre-separator) was sampled in the PBZ with a sampling tube using a length of 1 m.

One aethalometer measured in the PBZ of the worker, and the other measured in the BGZ. The filter strips in the aethalometers were replaced at least once per day to reduce the loading effects (Jimenez *et al.*, 2007; Kim *et al.*, 2016). During data evaluation the Aethalometer Optimized Noise-reduction Averaging (ONA) algorithm was used to reduce the occurrence of negative values to virtually zero while preserving the significant dynamic trends in the time series (Hagler *et al.*, 2011). The parameter delATN was set to 0.01. To enhance the accuracy of the eBC data and compensate for underestimation, a correction factor of 3.4 for the CNT material (Nanocyl) was applied to the aethalometer data, based on previous calibrations by Hashimoto *et al.* (2013). For WT 2, only CNTs from Nanocyl were used, while in WT 1 only CNTs from NanoLab were used. A combination of both types of CNTs were used in WTs 3–6. Since Hashimoto *et al.* (2013) did not present a correction factor for CNTs from NanoLab, we assumed the value 3.4 for all WTs.

##### Particle number concentration and size distribution

Two aerodynamic particle sizers (APS, model 3321, TSI Inc., USA) were used to measure number concentrations and size distributions of particles 0.5–20 µm based on aerodynamic equivalent particle size. A time-resolution of 5 s was used. One APS sampled from the EZ (0.1–0.2 m) of the CNT source, and the other from the BGZ. In the data evaluation, the difference in counting efficiency for 0.5–1 µm particles between the two APS instruments was adjusted for by calculating the concentration ratio (BGZ/EZ) between the two instruments from several in-field lunch calibration measurements during a time period with stable concentrations. The EZ values were then multiplied by these ratios.

A condensation particle counter (CPC, model 3775, TSI Inc., USA) was used to measure the particle number concentration (diameter >0.007 µm) in the EZ, with a time-resolution of 5 s.

A scanning mobility particle sizer (SMPS) consisting of a differential mobility analyser (DMA, model 3071, TSI Inc., USA) and a CPC (CPC, model 3010, TSI Inc., USA) was used to measure size distribution for particles 0.010–0.51 µm (mobility diameter) in the BGZ, with a time-resolution of 180 s.

The sampling inlets in the EZ consisted of three stainless steel tubes put together, with each tube connected to a direct-reading instrument via Tygon tubing. The inlets were placed as close as possible to the expected emission source, typically at 0.1–0.2 m distance. The sampling lines had a diameter of 6 mm and a total length of 1 m. Filter cassettes for EZ sampling were also positioned at the top of the instrument inlets.

#### Air sampling methods and analysis

##### Filter sampling of EC

Time-integrated samples of respirable and inhalable mass concentrations of EC were collected on quartz filters (37 and 25 mm, respectively, SKC Inc., USA) fitted in conductive three-piece filter cassettes (SureSeal, SKC Inc., USA) in the PBZ, EZ, and BGZ. Cyclones (BGI4L, BGI Inc., USA) were used for collection of the respirable fractions (50% cut-off at an aerodynamic equivalent particle diameter of 4 µm). Open-face sampling with 25 mm cassettes was used for collection of inhalable fractions, since this is considered to approximate the inhalable size fraction for fine, well dispersed powders (Dahm *et al.*, 2015). An Escort ELF pump (MSA, USA) provided sample flow rates set at 2.2 l min^−1^ for respirable sampling and 2.5 l min^−1^ for inhalable sampling. The air flow rates were regularly controlled with a primary calibrator (TSI Model 4100 Series, TSI Inc., USA).

All EC samples were analysed according to the National Institute for Occupational Safety and Health (NIOSH) NMAM 5040 protocol for thermal–optical analysis (DRI Model 2001 OC/EC Carbon Analyzer, Atmoslytic Inc., Calabasas, CA, USA) (Birch and Cary, 1996). The method was modified with an extended oxidation time at the highest temperature in order to achieve complete oxidation of all carbonaceous nanomaterials. Further details of the analytical procedure are given elsewhere (Hedmer *et al.*, 2014; Lovén *et al.*, 2021). The limit of detection (LOD) of the method for EC is 0.06 µg C cm^−2^, corresponding to 0.6 and 0.5 µg C m^−3^ for a 4-h sample for the respirable and inhalable fraction, respectively. During the analysis a high background was observed in the analytical instrument, including for the field blank filters. This was adjusted for by correcting the EC values by an average of the field blanks.

#### Filter sampling for SEM analysis

Time-integrated PBZ and EZ samples of respirable and inhalable fractions were collected on non-fibrous polycarbonate membrane filters with a pore size of 0.4 µm (37 and 25 mm, respectively, SKC Inc., USA), mounted in conductive three-piece filter cassettes. Cyclones (BGI4L, BGI Inc., USA) were used to collect respirable fractions of particles. Open-face sampling of 25 mm cassettes was conducted to collect inhalable fractions of particles. The air flow rate was set at 2.2 l min^−1^ for respirable sampling and 2.1 l min^−1^ for inhalable sampling, and was regularly controlled.

Personal and emission samples were analysed with SEM (FEI Nova NanoLab 600, FEI Company, USA) according to a previously described method (Hedmer *et al.*, 2014; Ludvigsson *et al.*, 2016). The LOD was determined to be between 0.13 and 2.5 cm^−3^ CNT-containing particles for 25 mm filter samples (inhalable fraction), and 14–20 areas per filter were randomly chosen for image acquisition. Each image area was 9050 µm^2^. The LOD was determined to be between 0.30 and 4.4 cm^−3^ CNTs for 37 mm filter samples (respirable fraction), and 18–21 areas per filter were randomly chosen for image acquisition. No CNTs were detected in the field blanks.

#### Surface sampling and SEM analysis

Based on a previously developed method for sampling of surface contamination with CNTs, different surfaces at the company were sampled with tape stripping (Hedmer *et al.*, 2015; Isaxon *et al.*, 2020; Lovén *et al.*, 2021). In short, adhesive tape was used to collect single tape samples from different surface locations related to the monitored WTs. The sampled surfaces were in both the near-field and far-field zone of the exposure sources. The sticky surface of the tape was pressed against the workplace surface to be sampled and then pulled off and placed with the sticky side down on a new sheet of plastic film. A new pair of nitrile gloves was used for each collected tape sample.

In total, 39 tape samples and 5 field blanks were collected from surfaces at the workplace. The sampled surfaces were made of metal, plastic, cardboard, enamelled concrete, laminating foil, clinker slab, and wood, and most of them (87%) were considered to have a smooth finish. Three sampled floors and two buttons were considered to have a rough surface finish. The characteristics of the sampled surfaces are presented in Supplementary Table S1 (available at *Annals of Work Exposures and Health* online). The tape samples were analysed with SEM according to a previously described method (Hedmer *et al.*, 2015). The LOD was determined to be >0.022 µm in size [one pixel], and >500 sample^−1^. No CNTs were detected in the field blanks.

##### Characterization of CNT raw materials

SEM analysis was carried out for the raw CNT materials, and the results are presented in Supplementary Table S2 (available at *Annals of Work Exposures and Health* online).

### Statistical analysis

Descriptive statistics for the emission and exposure data are presented as arithmetic mean with minimum (min) and maximum (max) values. The statistical analysis was performed with IBM SPSS Statistic 25 software for Windows. *P*–*P* plots indicated that EC and eBC data were approximately log-normally distributed. Spearman’s rank test was used to investigate the correlation between EC and eBC. Values below the LOD were given the value of half the LOD.

## Results

### Direct-reading instruments

#### Monitoring of eBC

**Figure 2.**
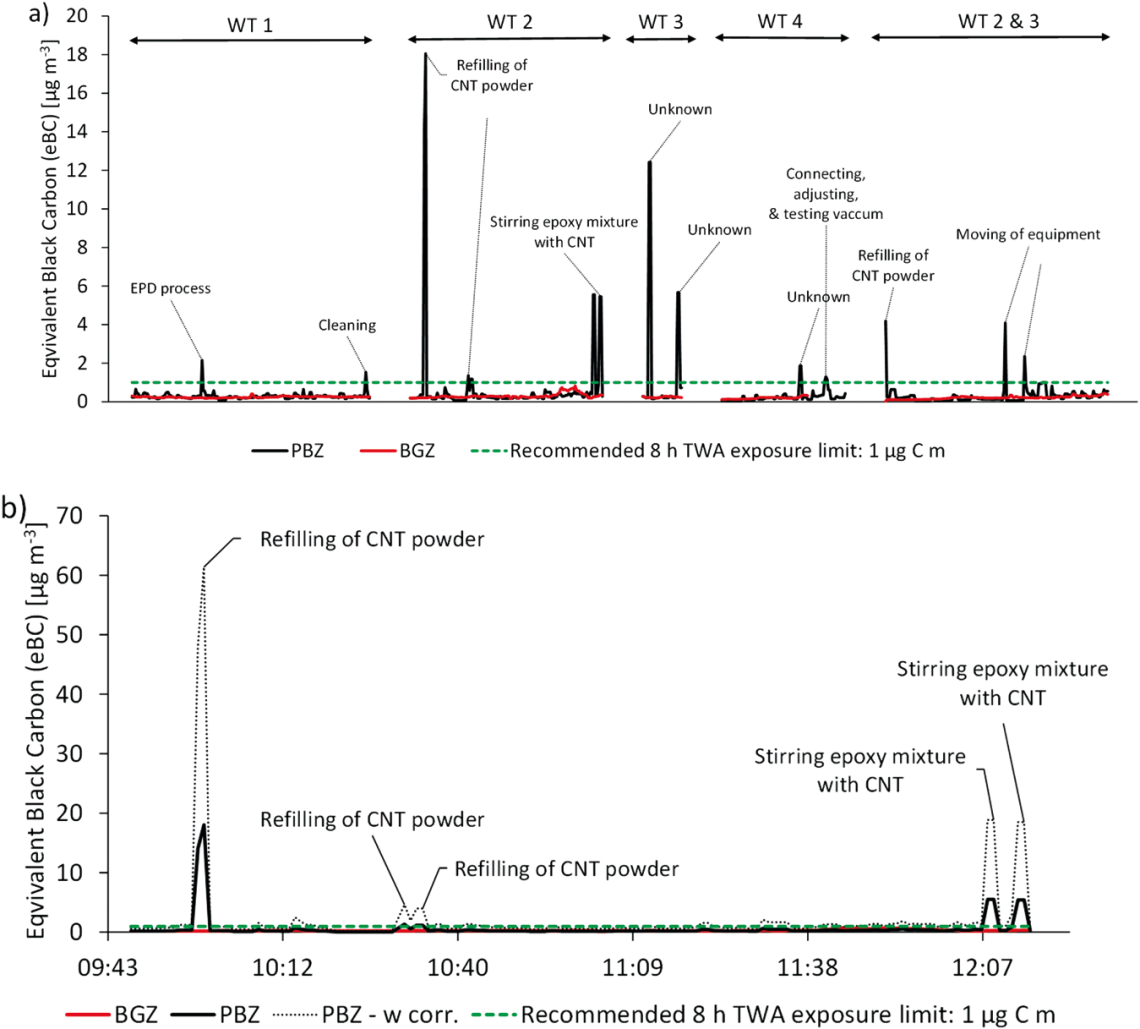
Time series of eBC measured by aethalometers. (a) For WTs 1–4 in the PBZ, and the BGZ. Peaks marked with ‘unknown’ could not be identified according to the logbook. WT 1: EPD, WT 2: direct mixing, WTs 3 and 4: vacuum infusion, WTs 2 + 3: direct mixing and vacuum infusion. (b) For WT 2 (direct mixing of epoxy with added CNTs) from online black carbon measurements in the PBZ, and the BGZ. The dotted line shows PBZ data corrected according to Hashimoto *et al.* (2013). The dashed line shows the exposure limit value recommended by NIOSH of 1 µg m^−3^ EC as a respirable mass 8-h time-weighted average (TWA) concentration. The *x*-axis shows continuous time.

The average eBC concentration for the different WTs is presented in Table 3, and the time-resolved measurements of eBC in the PBZ for four of the seven WTs can be seen in Fig. 2a. Unfortunately, no eBC results could be obtained for WTs 5 and 6 due to instrument failure during the PBZ measurements. Clear peaks above background were observed in terms of eBC mass concentration in the PBZ during WT 1: EPD process, cleaning (wiping off the treated composite product and packing it in bags), WT 2: refilling of CNT powder, stirring of epoxy mixture with CNTs, WT 4: connecting, adjusting, and testing of the vacuum, and WTs 2 and 3: refilling of CNT powder, moving of equipment. The majority of the peaks in Fig. 2a were identified on the basis of the notes in the logbook.

**Table 3.** Results from the filter-based measurements performed in seven WTs during production and testing of CNT composite. No respirable EC concentrations could be detected in any of the filters sampled in the EZ.

WT	Sampling time (min)	EZ conc.		PBZ conc.				BGZ conc.			
		Inhalable EC (µg C m^−3^)	CNTs detected via SEM^*a*^ (Yes/No)	eBC AM^*b*^/corrected^*c*^ AM (µg m^−3^)	Min–max/corrected min–max (µg m^−3^)	Inhalable EC (µg C m^−3^)	CNTs detected via SEM^*a*^ (Yes/No)	eBC AM (µg m^−3^)	Min–max (µg m^−3^)	Inhalable EC (µg C m^−3^)	CNTs detected via SEM^*a*^ (Yes/No)
Electrophoretic deposition (WT 1)	208	1.3	No	0.29/0.99	0.07–2.16/0.24–7.33	6.8	No	0.24	0.18–0.40	0.7	No
Direct mixing of CNT epoxy (WT 2)	122	<LOD^*d*^	No	0.65/2.21	0.07–18.1/0.25–61.4	0.8	**Yes (1.7)** ^*e*^	0.30	0.16–0.83	1.3	No
Vacuum infusion of CNT epoxy (WT 3)	20	9.8	No	1.39/4.74	0.16–12.4/0.53–42.2	<LOD	No	0.24	0.19–0.28	<LOD	No
Preparation and vacuum infusion of epoxy (WT 4)	67	<LOD/15.9^*f*^	No	0.26/0.88	0.04–1.87/0.14–6.34	5.1	No	0.19	0.12–0.34	3.4	**Yes (0.6)** ^*e*^
Direct mixing of CNT epoxy and vacuum infusion (WTs 2 and 3)	179	<LOD	No	0.31/1.06	0.04–4.19/0.13–14.3	3.0	No	0.21	0.03–0.45	5.9	No
Power sawing (WT 5)	14	429	No	—^*g*^		<LOD	No	—	—	9.0	No
Short-beam shear testing (WT 6)	30	<LOD	No	—		<LOD	No	—	—	<LOD	No
Cleaning (WT 7)	89	—	—	—		4.7	—	—	—	—	—

^*a*^SEM analysis of filter.

^*b*^Arithmetic mean.

^*c*^Corrected according to Hashimoto *et al.* (2013).

^*d*^Not detected.

^*e*^Number of agglomerates per cm^3^.

^*f*^Measured close to the air outlet of the infusion pump.

^*g*^Not measured due to instrument failure.

**Figure 3.**
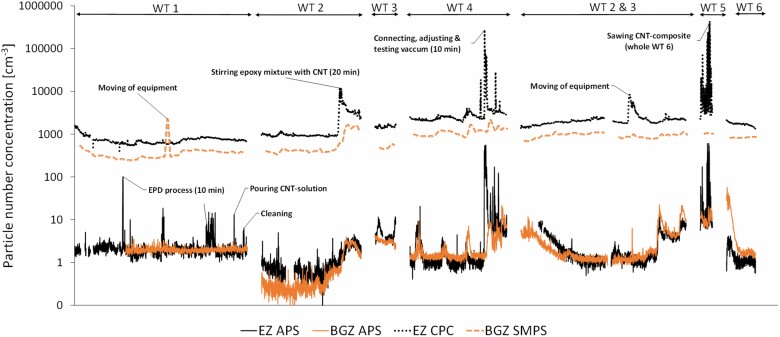
Time series from direct-reading instruments (APS, CPC, and SMPS) measured in the EZ and BGZ. All instruments measure the total particle concentration. Measurements were performed for all six WTs. WT 1: EPD, WT 2: direct mixing, WTs 3 and 4: vacuum infusion, WTs 2 + 3: direct mixing and vacuum infusion, WT 5: power sawing, WT 6: short-beam shear testing. The *x*-axis shows continuous time.

Strong eBC mass concentration peaks were seen, for example during refilling of the CNT powder (WT 2 and WTs 2 and 3), which was handled openly. This refilling took place in the storage room, which is why no corresponding peaks could be seen in showing the stationary measurements (Fig. 3), as the APS and CPC used for these measurements were placed in the chemical laboratory. The highest peak occurred during WT 2 when the worker openly transferred CNT powder from the bulk container into a small plastic container in the storage room (Fig. 2b). The other peaks also showed increased exposures to eBC during mixing and stirring of CNT epoxy resin in WT 2. The eBC concentration measured in the PBZ was corrected according to the calibration by Hashimoto *et al.* (2013) and is also presented in Fig. 2b.

WT 2 was performed twice, and the second time the eBC mass concentrations were lower. In WT 3, the first peak in Fig. 2a may relate to the opening of the door to the heated oven, and the second eBC peak may relate to the start of the vacuum pump.

No other clear sources of EC were identified at the company. In general, the background levels of eBC were consistently low (Table 3) and the average background eBC concentration of all WTs was 0.24 ± 0.04 µg m^−3^. The low background eBC could originate from, for example, carbonaceous contamination (e.g. CNTs) in the facility or from external sources (e.g. soot). Airborne CNTs were detected on a filter sample collected in the BGZ (see Table 3). The background eBC concentrations were well below the NIOSH recommended exposure limit (REL) of CNTs based on EC (1 µg C m^−3^ for a full 8 h shift). The average PBZ eBC concentration of all WTs was 0.58 ± 1.17 µg m^−3^, which is more than twice the background concentration. Moreover, clear eBC peaks above background were observed indicating that CNTs were emitted during the production of CNT composite. The average corrected eBC concentration in the PBZs was 1.98 ± 3.99 µg m^−3^. Compared with the NIOSH REL for EC, the occupational exposure (measured in the PBZ) to CNTs measured as uncorrected eBC was on average ~60% of the REL, and the corrected eBC was on average ~200%.

#### Particle number and size distribution

The mean particle number concentrations from the emission measurements with APS and CPC are presented in Table 4. Power sawing (WT 5) was the WT that emitted the highest particle number concentration in the EZ of both nanoparticles and coarse particles followed by vacuum infusion of epoxy (WT 4). Fig. 3 presents particle number concentrations for six WTs. Clear peaks above background were observed in terms of number concentration in the size range 0.5–20 µm (APS measurements) during WT 1: the EPD process, moving of equipment, pouring of CNT solution from the EPD vessel into a bucket, cleaning (wiping off the treated composite product and packing it in bags), WT 4: connecting, adjusting, and testing of the vacuum, and WT 5: sawing of the CNT composites. At the beginning of WT 6, the APS measurements showed a relatively high concentration peak for the BGZ. The only explanation we can find for this large peak is that the notes state that some movements were going on at the background station. At the end of WTs 2 and 3, another worker performed without warning powder coating at another work station right next to the background measuring station, which could explain the higher APS peaks in the BGZ than in the EZ during this time.

**Table 4.** Emission concentrations from the direct reading instruments (APS and CPC).

WT	Particle number concentration (cm^−3^)									
	1–10 µm in EZ		1–10 µm in BGZ		0.5–1 µm in EZ		0.5–1 µm in BGZ		0.007–0.5 µm in EZ	
	AM^*a*^	Min–max	AM	Min–max	AM	Min–max	AM	Min–max	AM	Min–max
Electrophoretic deposition (WT 1)	0.52	0.11–18.1	0.29	0.06–1.36	2.41	0.72–99.3	1.72	1.15–2.53	757	401–2049
Direct mixing of CNT epoxy (WT 2)	0.26	0.02–2.23	0.11	0.00–0.65	0.85	0.00–3.66	0.63	0.05–3.50	1602	782–12 467
Vacuum infusion of CNT epoxy (WT 3)	0.27	0.00–0.56	0.15	0.04–0.31	4.73	2.36–11.2	2.96	1.88–4.13	1383	892–2026
Preparation and vacuum infusion of epoxy (WT 4)	0.88	0.02–69.7	0.17	0.01–0.96	12.8	0.51–660	2.64	0.89–20.8	4034	1887–273 193
Direct mixing of CNT epoxy and vacuum infusion (WTs 2 and 3)	0.49	0.00–4.18	0.54	0.04–24.1	3.03	0.00–16.5	2.75	0.58–19.1	2150	1367–8530
Power sawing (WT 5)	2.84	0.00–139	1.04	0.38–2.42	15.6	0.00–556	10.6	5.59–20.3	5705	744–441 782
Short-beam shear testing (WT 6)	0.14	0.00–3.07	0.34	0.04–6.11	1.53	0.00–5.03	6.88	1.08–58.0	1678	0–11 039

^*a*^Arithmetic mean.

For particles larger than 0.007 µm (CPC measurements) clear peaks above background were seen during the following events: WT 2: stirring of epoxy mixture with CNTs, WT 4: connecting, adjusting, and testing of the vacuum, WTs 2 and 3: moving of equipment a second time (with no corresponding peak for the APS), and WT 5: power sawing of the CNT composites.

### Filter-based sampling

**Figure 4.**
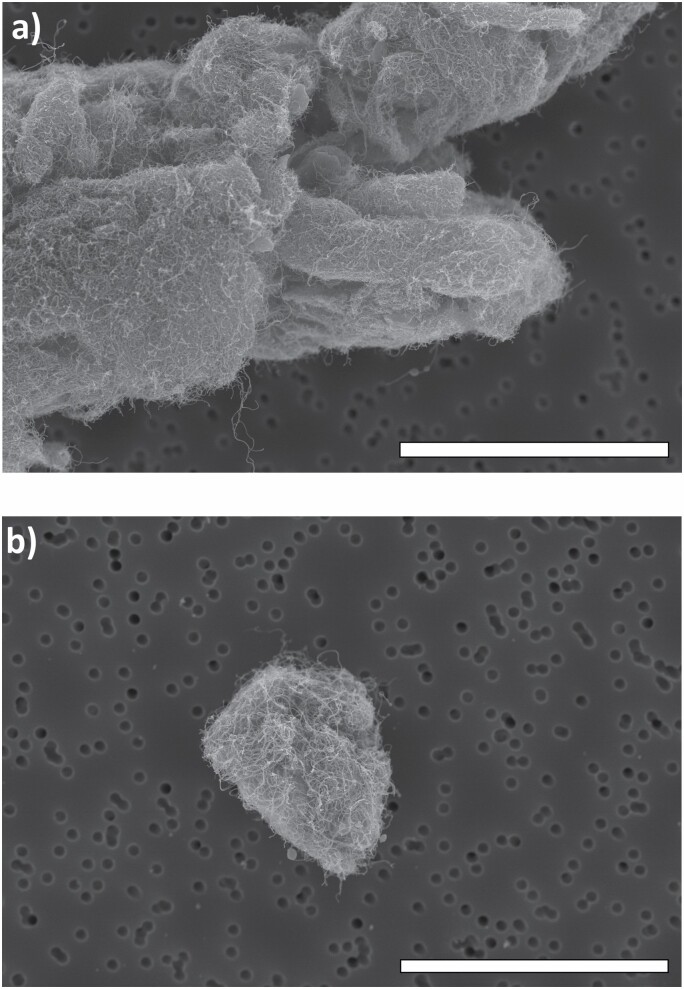
Airborne CNTs detected during mixing of CNT powder with epoxy in the production of nanocomposite. The samples (a and b) were collected in the PBZ. The scale bar in each image equals 1 µm.

The results from the filter-based emission and exposure measurements are presented in Table 3. Airborne CNTs were identified in two of the filter samples analysed with SEM (inhalable fractions). One was sampled in the PBZ of worker B during direct mixing of CNT epoxy (WT 2) and the other was sampled in the BGZ at preparation and vacuum infusion of epoxy (WT 4). During WT 2, the worker openly transferred CNT powder from the bulk container into a small plastic container in the storage room. The particle number concentration was counted to approximately 1.7 agglomerates cm^−3^ in the filter sample. Ludvigsson *et al.* (2016) defined three classes of airborne CNT-containing particles: type 1: particles with aspect ratio length:width >3:1 (fibrous particles); type 2: particles without fibre characteristics but with high CNT content; and type 3: particles with visible embedded CNTs. The detected and dominant airborne CNT-containing particles in the study were large agglomerates which looked like ‘porous balls’ and could be classified as type 3 according to Ludvigsson *et al.* (2016). No free individual CNTs were revealed in the SEM analysis. Typical images of airborne CNTs from WT 2 can be seen in Fig. 4. The SEM analysis of the CNT raw materials showed similar porous balls of CNTs.

Inhalable concentrations of EC were detected in five of eight PBZ filter samples (Table 3). The highest EC concentrations were detected during WT 1 (EPD) and WT 4 (preparation and vacuum infusion of epoxy). In the EZ, inhalable concentrations of EC above the LOD were detected in four of seven filter samples. The highest emission occurred during power sawing (429 µg m^−3^, WT 5), but EC was also emitted at the air outlet from the infusion pump used for the vacuum infusion during WT 4, and vacuum infusion of CNT epoxy (WT 3). Inhalable EC concentrations were also detected in the BGZ during five of the WTs (1, 2, 4, 2 and 3, and 5). In two of the WTs (2, and 2 and 3) the concentrations of EC in the BGZ were higher than in the EZs. No respirable EC concentrations could be detected in any of the filters sampled in the EZ. The statistical analysis showed no significant correlation between EC and eBC. A correlation plot between EC and eBC is presented in Supplementary Fig. S2 (available at *Annals of Work Exposures and Health* online).

### Surface contamination

**Figure 5.**
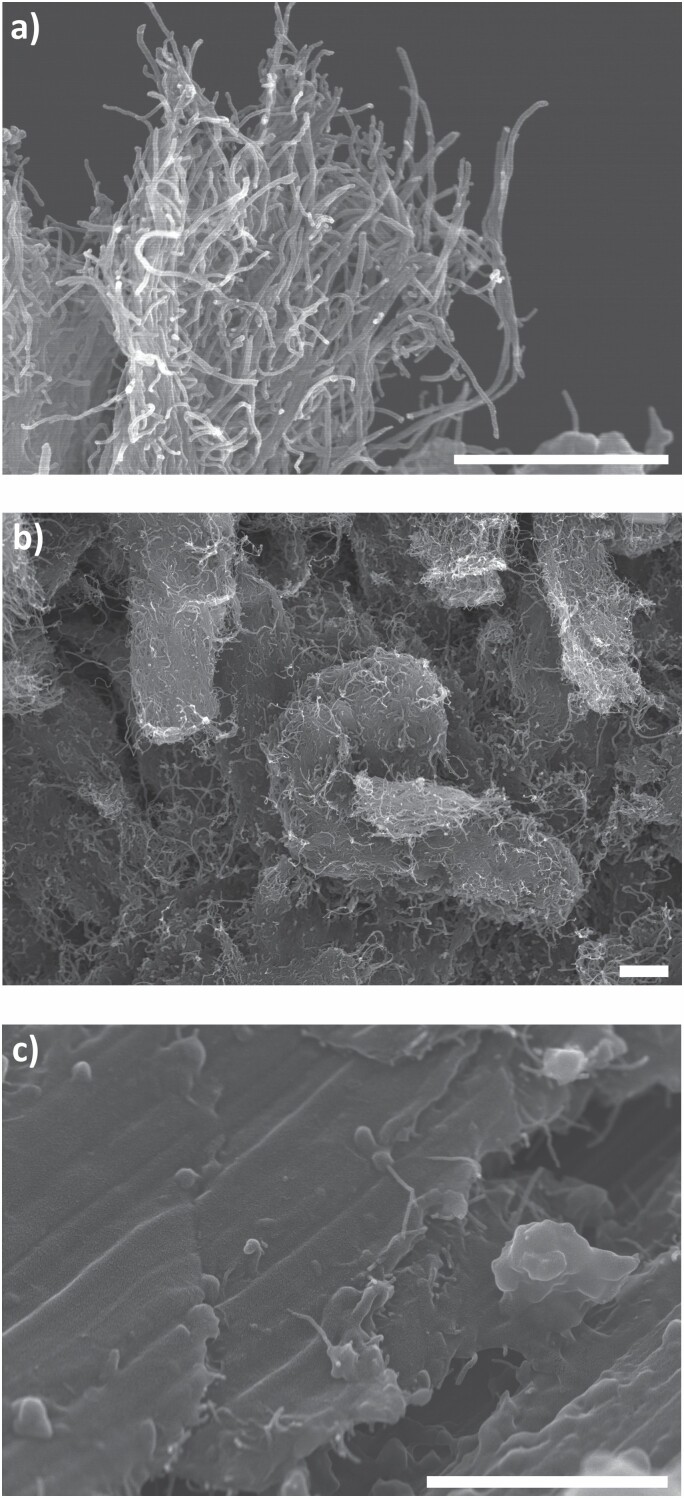
SEM images of surface contamination of CNTs (a) detected as surface contamination on the storage shelf at the chemical laboratory, (b) detected as surface contamination on the balance display at the chemical laboratory, and (c) detected as surface contamination on the work bench for the saw in the manufacturing laboratory. The scale bar in each image equals 1 µm.

Workplace surfaces in the chemical and manufacturing laboratories, dressing room, corridor, and offices were sampled with the tape stripping method. The results from the SEM analysis of the tape samples are presented in Supplementary Table S1 (available at *Annals of Work Exposures and Health* online). CNTs were detected in 21% (*N* = 8) of the 39 samples: four surfaces in the chemical laboratory, three surfaces in the manufacturing laboratory, and handle on the corridor side of the door between the chemical laboratory and the corridor (see Fig. 1). Seven of the eight tape samples were collected in the near-field zones of the WTs, and only one was collected in the far-field zone (door handle). SEM images of CNTs as surface contamination can be seen in Fig. 5. CNTs were present on the workplace surfaces in the chemistry laboratory as large heterogeneous agglomerates. Elongated CNT-like features were also detected in the sawdust on the work bench after power sawing of CNT composite (Fig. 5c). It is possible that CNTs embedded in the epoxy matrix became visible in the SEM images.

## Discussion

### Aerosol data

To our knowledge, this is the first published study using a portable aethalometer in the PBZ to monitor occupational exposure to CNTs. With the aethalometer, time-resolved eBC exposure data were obtained for different WTs, and peak exposures during different WTs could be identified. For example, from the logbook we have verified that dry CNT powder was openly handled at the time when the strongest eBC peaks in the PBZ were registered during WT 2 (first measurement). In the SEM sample also collected in the PBZ during the same WT, large agglomerates of CNTs (1.7 cm^−3^; shortest particle diameter approx. 2–20 µm) could be identified, which strengthens the eBC data. The 2 h EC sample (inhalable fraction) was 0.8 µg m^−3^ in the sample collected simultaneously in the PBZ during the same WT, while the sample collected in the EZ was below LOD. During WT 2 (second measurement), an eBC peak exposure in the PBZ also occurred when CNT powder was handled, but it was approximately four times smaller than the peak during the first measurement of WT 2. During this second measurement of WT 2, an inhalable EC concentration of 3.0 µg m^−3^ was found in the PBZ. However, SEM analysis did not identify any agglomerates of CNTs on the filter sample collected simultaneously from the PBZ. EC was also measured in the PBZ (6.8 µg m^−3^) and in the EZ (1.3 µg m^−3^) during WT 1, but the SEM samples from the EZ and PBZ do not prove that the measured EC concentration was derived from CNTs.

The filter sampling of EC close to the air outlet of the infusion pump used for vacuum infusion, showed that EC was present in the outgoing air. The sampled air came from the vacuum system and had passed through the CNT-coated fabrics, and thus the detected EC may have originated from that CNT source.

Concentrations of both EC and eBC were also measured in the BGZ for some of the WTs, possibly originating from resuspension of surface contamination containing EC (e.g. CNTs) in the laboratories due to human movement during the sampling campaign (Schneider et al., 1999, 2000). According to Hashimoto *et al.* (2013), eBC tends to underestimate the CNT mass concentrations, and so a correction factor of 3.4 was used in this study to achieve more realistic CNT concentrations, in line with the figure reported for Nanocyl CNT (Hashimoto *et al.*, 2013). The exposure peak at the beginning of WT 2 generated from refilling of CNT powder increased from a maximum value of ~18 to ~61 µg m^−3^ at a 1 min time-resolution. The eBC peaks in WT 2 may impose health risks for the workers, as they represent short but high exposures to CNTs and we know that dry CNT powder was openly handled. Additionally, the corresponding SEM sample showed an estimated concentration of 1.7 agglomerates cm^−3^, and the personal EC concentration in WTs 2 and 3 (3.0 µg m^−3^) was above 1 µg m^−3^ (NIOSH REL); these results confirm the exposure situation. The actual fibre concentration in the sample was much higher, but it was not possible to count all individual CNTs in the agglomerates (Fig. 4). A review by Debia *et al.* (2016) confirmed occupational exposure to airborne CNTs in 52 exposure situations, mainly related to handling tasks such as pouring, weighing, mixing, and sanding of CNT-containing composites, and involving CNTs in powder form, liquid suspension, or embedded in a matrix. Similar findings are presented in this study.

Infiltrated particles from ambient air could contribute to the workplace eBC aerosol. However, the nearby rural background of the facility had the typical extent of EC <1 µg m^−3^. Our background measurement of eBC showed a low level of eBC, and so the extent of anthropogenic sources of infiltrated EC/eBC contamination inside the facility was low.

Many of the peaks measured with the portable aethalometer in the PBZ could also be seen in the aerosol data from the CPC and APS measurements in the EZ. Based on the results from the workplace measurements in the study it was not possible to conclude that eBC is a good proxy of EC. However, the time-resolved data generated with the aethalometer gave information on which of the steps in the different WTs were associated with the release of CNTs. These measurements therefore show the value of aethalometers for monitoring of CNT exposure. As the devices are user-friendly in field work and relatively cheap, they are a particularly promising tool to use in a first exposure assessment according to Tier 2 of the guidelines published by the Organisation for Economic Co-operation and Development (2015). They also provide complementary information to time-integrated filter collection by linking exposures to specific WTs.

During the handling (scooping, weighing, mixing) of dry CNT powder in WT 2, CNTs became airborne and were detected in the PBZ. In a previous study by Cena and Peters (2011), no CNT particles were found to be emitted during the CNT powder weighing process in production of CNT composite. Thompson *et al.* (2015) found no CNT in the PBZ at a downstream user of polymer nanocomposites. However, large agglomerates of CNTs were identified in the workplace air through area sampling.

When the CNT composite material was sawn during WT 5, clear peaks of both nanoparticles (CPC measurement) and coarse particles (APS measurement) were seen. High concentrations of nanoparticles (0.007–0.5 µm) were released for each sawing cycle, with mean particle number concentration of ~5700 cm^−3^. The corresponding concentrations for coarser particles (0.5–1 and 1–10 µm) were ~16 and 3 cm^−3^, respectively. A high EC concentration (429 µg m^−3^, inhalable fraction) was also measured in the EZ. Ding *et al.* (2017), who performed measurements in the EZ during sawing of CNT composite based on polyurethane, reported much lower mean particle number concentrations of ~57 cm^−3^ in the size range of 0.01–0.3 µm, and ~1.3 cm^−3^ in the size range 0.25–32 µm. Another study involving sawing of polyurethane composite with CNTs measured much higher number concentrations of up to ~28 700 for small particles (range 0.01–0.3 µm) near the emission source (Kuijpers *et al.*, 2019). In a study by Ogura *et al.* (2019), portable aethalometers detected increased eBC concentrations close to the source during the sawing process, while the other aerosol instruments (CPC: 0.01–3 µm and an optical particle sizer: 0.3–10 µm) did not respond with any clear peaks. In the current study, eBC data were unfortunately missing for the sawing process due to technical issues. In the filter sample collected during sawing, SEM analysis did not detect any individual CNTs, agglomerates, or aggregates among the collected particles. Similar results were also presented by Kuijpers *et al.* (2019) while Ding *et al.* (2017) reported elongated features matching the CNTs that were used. Ogura *et al.* (2019) detected CNTs in aerosol particles generated by sawing at a distance corresponding to that between an operator’s breathing zone and the cutting part.

### Surface contamination

Surface contamination of CNTs was found on several surfaces in the chemical and manufacturing laboratories. Even though CNT powder was handled openly in the chemical and manufacturing laboratories, surface contamination could only be detected on one surface (a door handle on the corridor side) outside these laboratories. The percentage of surface samples with CNT contamination was in the same range as reported by Lovén *et al.* (2021), in which TiO_2_ nanofibres were tape sampled, but lower than reported in a previous study of CNTs (Hedmer *et al.*, 2015). The detected surface contamination could most likely be related to the different production steps of the nanocomposite as most of the contaminated surfaces were found in the near-field zone in the chemical and manufacturing laboratories, where the WTs were performed. However, contamination was detected on one fair-field surface which could indicate CNT contamination in other parts of the facility. The surface contamination of CNTs could potentially be resuspended into the workplace air, which would pose a risk of secondary inhalation exposure. For example, agglomerates of CNTs were detected in a SEM sample collected in the BGZ during WT 4 (~0.6 agglomerates cm^−3^). These might have originated from surface contamination in the laboratories that had been resuspended into the workplace air by human indoor work activities, such as walking or moving (Hedmer *et al.*, 2015).

During power sawing of the CNT composite, an aerosol containing CNTs was generated. Sawing was openly performed in the manufacturing laboratory, but with a local exhaust ventilation placed close to the emission source. However, not all generated particles were ventilated out, as demonstrated by the surface contamination of CNT-containing particles on the work bench. This surface contamination might have the potential to be resuspended into the workplace air.

### Limitations

Field measurements at real workplaces can sometimes be challenging, due to the complexity that come from several different processes being performed at the same time in the same facility. An example is WTs 2 and 3 in the present study, where a powder coating process was unexpectedly carried out close to the BGZ measuring station, influencing the aerosol data. This occurred despite our prior agreement with the company that no other aerosol generating processes would be performed during the measuring campaign.

Conditions in this real workplace study included low emitted concentrations in combination with short sampling times and the presence of complex nanomaterial with CNTs occasionally embedded in composites. This made the concentrations more uncertain and increased the possibility of contradictory results. Some of the WTs were short, and thus the sampling times in the PBZs and EZs were also short (14–30 min). This is of course a limitation, and these concentrations may be more uncertain than those with longer sampling times. For particle collection on filters it can also be difficult to obtain a sufficiently large sample amount for the analysis in a short period of time.

In some of the WTs the CNT release was transient and short lived. In future measurements of eBC a higher time-resolution should be considered to get more detailed exposure data. It was also difficult to define some of the WTs. Personal direct-reading instrument showed eBC exposure between the WTs. It seems that there might have been diffuse sources of carbonaceous particles (e.g. CNT contamination) at the site, since eBC peaks of unknown origin were measured during WT 3. If the study had been performed over a longer period of time it might have been possible to distinguish the different WTs from each other and obtain a better statistical selection for the typical exposures for the different WTs.

The aethalometers measured the total fraction of airborne particles. Thus, the measured eBC concentrations would have been more reliable if a respirable dust cyclone had been connected to the inlet of the aethalometer. However, no respirable EC concentrations were measured in the EZ for any of the WTs.

Another limitation, according to the literature, is the eBC concentration measured with an aethalometers could underestimate the CNT mass concentration, depending on the type of CNT and the size of aerosolized CNT particles (Hashimoto *et al.*, 2013; Kim *et al.*, 2016). According to Hashimoto *et al.* (2013), it may also be difficult to detect low-level CNTs comparable to the lower REL for EC (the NIOSH proposal of 1 µg m^−3^) in the presence of background aerosols. In our study, transport losses of coarser CNT-containing particles within the sampling tube (1 m) and internal losses in the aethalometer may further explain why there was no significant correlation between EC and eBC. This is one of the first applications of the micro-aethalometer to measure PBZ exposures containing significantly larger particles. There are to our knowledge no studies available of sampling losses of larger particles in this instrument, but clearly there is a need for such studies.

An alternative monitoring method of airborne CNTs could have been elemental analysis of catalytic metals in the CNTs collected during filter sampling (Maynard *et al.*, 2004; Kato *et al.*, 2017). Analysis in the previously mentioned studies showed that the CNTs contained traces of different metal, such as Fe, Mo, Al, and Co. Thus, filter sampling of catalysts could have been used as a surrogate for total CNT mass concentration.

## Conclusion

Characterization of a workplace producing CNT composite using real-time aerosol instruments showed that weighing and mixing of dry CNT powder material generated the highest emissions and exposure to CNT-containing particles. Power sawing of CNT composite material also released high concentrations of particles, but no individual CNTs, agglomerates, or aggregates were detected on filter samples using SEM analysis.

This study also shows the feasibility of using a portable direct-reading aethalometer in the PBZ to monitor the occupational exposure to CNTs. The instrument provided time-resolved eBC exposure data to complement the time-integrated filter samples, and allowed us to identify particle peak exposures from specific parts of the WT. Thus, aethalometers could be used for CNT exposure monitoring as a complement to time-integrated filter sampling of EC used for quantitative exposure assessment. However, it was not possible to conclude that eBC is a good proxy for EC. The eBC data were corrected using a correction factor to reduce the underestimation of CNT concentration.

Surface contamination of CNTs was found on several surfaces at this downstream user, most likely originating from the monitored WTs, as most of the contaminated surfaces were found in the near-field zone. Elongated CNT-like features were detected as surface contamination on the work bench close to the saw after sawing of CNT composite. This surface contamination could potentially be resuspended into the workplace air, and may pose a risk of secondary inhalation exposure since half-face respirators were not constantly used. In conclusion, despite the use of different protective equipment and engineering controls at the workplace there was a risk of harmful exposure to airborne CNTs due to open handling of CNT powder during some WTs.

## Supplementary Material

wxac015_suppl_Supplementary_Material

## Data Availability

The data underlying this article will be shared on reasonable request to the corresponding author.
