# Supplementary material for: Real-Time Emission and Exposure Measurements of Multi-walled Carbon Nanotubes during Production, Power Sawing, and Testing of Epoxy-Based Nanocomposites
Source: Ann Work Expo Health. 2022 Mar 17;66(7):878–94. doi: 10.1093/annweh/wxac015 (PMC9357347; doi:10.1093/annweh/wxac015)
Supplement: wxac015_suppl_Supplementary_Material [file wxac015_suppl_supplementary_material.pdf]

Supplementary materials

Real-time Emission and Exposure Measurements of Multi-walled Carbon Nanotubes during Production, Power Sawing and Testing of Epoxy-based Nanocomposites

Maria Hedmer[1,2,3*], Karin Lovén[1,4], Johan Martinsson[5], Maria E. Messing[1,6], Anders Gudmundsson[1,4], Joakim Pagels[1,4]

[1]NanoLund, Lund University, P.O. Box 118, 22100 Lund, Sweden; [2]Occupational and Environmental Medicine, Department of Laboratory Medicine, Lund University, P.O. Box 117, SE-22100, Lund, Sweden; [3]Department of Occupational and Environmental Medicine, Region Skåne, SE-22381, Lund, Sweden; [4]Ergonomics and Aerosol Technology, Department of Design Sciences, Lund University, P.O. Box 118, SE-22100, Lund, Sweden; [5]Medical Radiation Physics, Department of Translational Medicine, Lund University, P.O. Box 117, SE-22100 Lund, Sweden; [6]Solid State Physics, Department of Physics, Lund University, P.O. Box 118, SE-22100, Lund, Sweden

*Author to whom correspondence should be addressed. Tel: +46-46173193; e-mail: maria.hedmer@med.lu.se

Table S1. Overview of the tape-sampled surface locations and the presence of CNTs on surfaces.

Number	Sampling location	Unit	Related to work task no.	Description of the sampled surfaces/distance to the expected exposure source	Surface characteristics		CNTs detected via SEM (Yes/No)
					**Material**	**Assessed indication of roughness**	
1	**Work areas**	Chemical laboratory	1	Work area next to fume hood A/near-field zone	Laminate foil	Smooth	No
2		Chemical laboratory	1	Work area inside fume hood A/near-field zone	Protective sheet with Al layer	Smooth	**Yes**
3		Chemical laboratory	2	Work area next to the fume hood/ far-field zone	Laminate foil	Smooth	No
4		Chemical laboratory	2	Work area inside fume hood B/ near-field zone	Protective sheet with Al layer	Smooth	No
5		Manufacturing laboratory	5	Work bench ~10 cm from the circular saw blade/near-field zone	Metal	Smooth	**Yes**
6		Manufacturing laboratory	5	Work bench ~10 cm from the circular saw blade, other side compared to no. 5/near-field zone	Metal	Smooth	**Yes**
7		Manufacturing laboratory	3	Work area next to the oven/far-field zone	Metal	Smooth	No
8		Manufacturing laboratory	3	Work area next to the oven/near-field zone	Protective sheet of cardboard	Smooth	No
9	**Floors**	Chemical laboratory	1	Floor next to the fume hood, covered with protective sheet/near-field zone	Protective sheet with Al layer	Smooth	No
10		Chemical laboratory	1	Floor at fume hood A with EPD process/near-field zone	Plastic flooring	Smooth	No
11		Chemical laboratory	2	Floor at the fume hood B with sonicator/near-field zone	Plastic flooring	Smooth	No
12		Manufacturing laboratory	5	Floor below the saw/near-field zone	Plastic flooring	Smooth	
13		Dressing room	-	Floor at the door opening between chemical laboratory and dressing room/far-field zone	Plastic flooring	Rough	No

Number	Sampling location	Unit	Related to work task no.	Description of the sampled surfaces/distance to the expected exposure source	Surface characteristics		CNTs detected via SEM (Yes/No)
					Material	Assessed indication of roughness	
14		Dressing room	-	Floor at a locker belonging to one of the CNT workers/far-field zone	Plastic flooring	Smooth	No
15		Storage room	2	Floor close to the storage of CNTs/near-field zone	Epoxy coated concrete	Rough	No
16		Storage room	2	Floor close to the storage of CNTs/near-field zone	Epoxy coated concrete	Rough	**Yes**
17		Manufacturing laboratory	6	Floor next to the short-beam shear testing equipment/near-field zone	Plastic flooring	Smooth	No
18		Corridor	-	Floor outside the chemical laboratory/far-field zone	Tiled flooring	Smooth	No
19		Office	-	Floor under a desk belonging to one of the CNT workers /far-field zone	Plastic flooring	Smooth	No
20	**Handles**	Door between dressing room and chemical laboratory	-	Door handle on the laboratory side/far-field zone			No
21		Chemical laboratory	1, 4	Handle of fume hood A/near-field zone	Metal	Smooth	No
22		Manufacturing laboratory	3	Handle to waste can at the EPD oven/near-field zone	Plastic	Smooth	No
23		Manufacturing laboratory	6	Computer mouse used with the short-beam shear testing equipment/far-field zone	Plastic	Smooth	No
24		Manufacturing laboratory	-	Handle of a fireproof door/far-field zone	Metal	Smooth	No
25		Door between corridor and chemical laboratory	-	Door handle on the corridor side/far-field zone	Metal	Smooth	**Yes**
26		Office	-	Computer mouse belonging to one of the CNT workers/far-field zone	Plastic	Smooth	No
27	**Other surfaces**	Chemical laboratory	1	Alarm button at fume hood A/near-field zone	Plastic	Smooth	**Yes**
28		Chemical laboratory	2	Top of a cupboard located next to fume hood B/far-field zone	Laminate foil	Smooth	No
29		Chemical laboratory	2	Storage shelf under fume hood B/near-field zone	Laminate foil	Smooth	**Yes**

Number	Sampling location	Unit	Related to work task no.	Description of the sampled surfaces/distance to the expected exposure source	Surface characteristics		CNTs detected via SEM (Yes/No)
					Material	Assessed indication of roughness	
30		Chemical laboratory	2	Waste bin/far-field zone	Metal	Smooth	No
31		Chemical laboratory	2	Balance display used in fume hood B for weighing CNTs/near-field zone	Plastic	Smooth	**Yes**
32		Chemical laboratory	1	Storage shelf under fume hood A/near-field zone	Laminate foil	Smooth	No
33		Manufacturing laboratory	5	Saw start button/near-field zone	Plastic	Rifled	No
34		Manufacturing laboratory	5	Saw stop button/near-field zone	Plastic	Rifled	No
35		Manufacturing laboratory	2	Plastic bag enveloping a CNT container/near-field zone	Plastic	Smooth	No
36		Manufacturing laboratory	5	Local extract ventilation/near-field zone	Metal	Smooth	No
37		Manufacturing laboratory	6	Short-beam shear testing equipment/near-field zone	Metal	Smooth	No
38		Manufacturing laboratory	5	Top of a cupboard 4 m away from the saw/far-field zone	Metal	Smooth	No
39		Manufacturing laboratory	2, 5	Top of a cupboard/far-field zone	Metal	Smooth	No

Table S2. Characteristics of the CNT materials.

Type of material	Producer	Diameter (nm)	Trace metals
COOH functionalized multi-walled CNT (powder)	NanoLab, USA	19.7 ± 8	Fe, Mo, Al, (Zr), (Au),(S)
COOH functionalized multi-walled CNT (in suspension)	NanoLab, USA	19.7 ± 8	Fe, Mo, Al, (Zr), (Au),(S)
Multi-walled CNT (powder)	Nanocyl, Belgium	12 ± 3	Al, Fe, Co, (Au), (Zr)

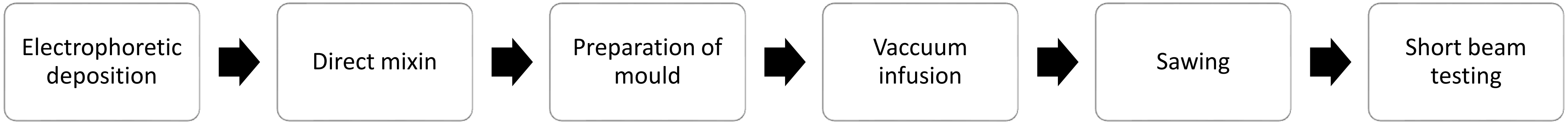

Fig. S1. Schematic overview of CNT composite production at the manufacturer.

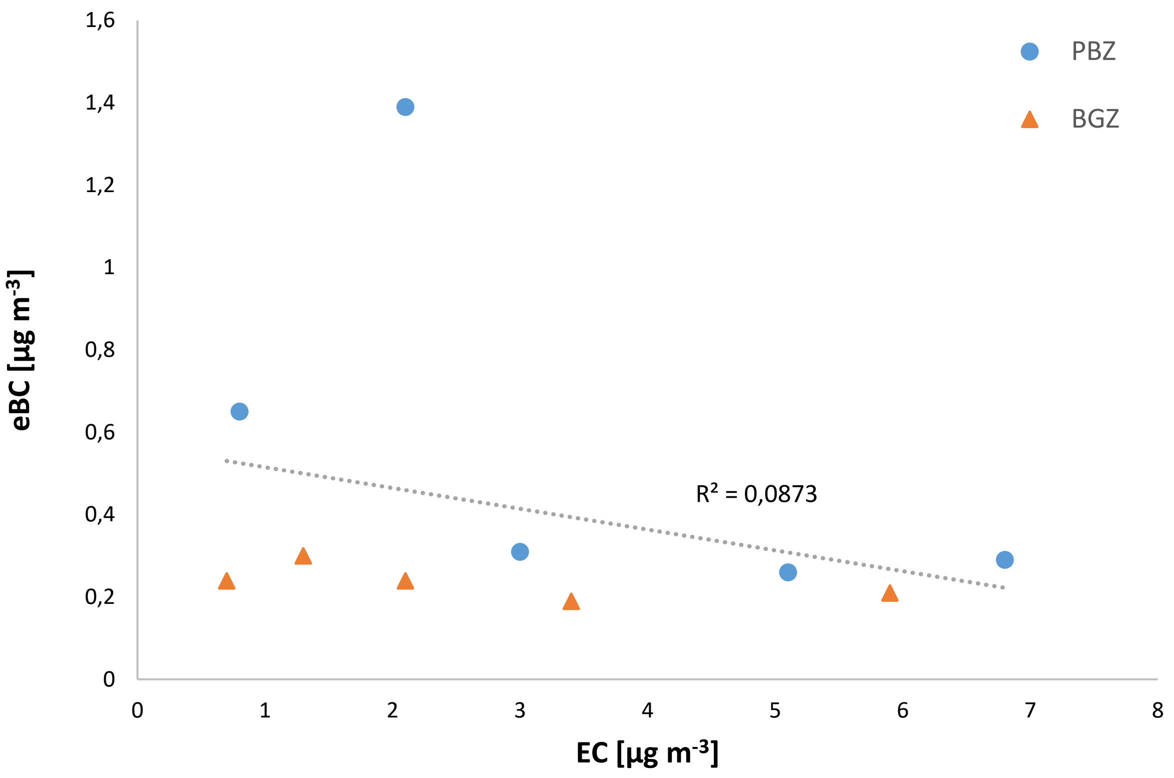

Figure S2. Correlation between elemental carbon (EC) and equivalent black carbon (eBC) in the personal breathing zone (PBZ) and background zone (BGZ). The Pearson correlation coefficient was 0.30 with linear regression. Non-detect samples were given the value of half the detection limit.
